# Urine and vaginal microbiota compositions of postmenopausal and premenopausal women differ regardless of recurrent urinary tract infection and renal transplant status

**DOI:** 10.1038/s41598-022-06646-1

**Published:** 2022-02-17

**Authors:** Floor Hugenholtz, Charlotte van der Veer, Matty L. Terpstra, Hanneke Borgdorff, Robin van Houdt, Sylvia Bruisten, Suzanne E. Geerlings, Janneke H. H. M. van de Wijgert

**Affiliations:** 1 Center for Experimental and Molecular Medicine, Amsterdam University Medical Centers location AMC, Amsterdam, The Netherlands; 2 Public Health Laboratory, Public Health Service of Amsterdam (GGD), Amsterdam, The Netherlands; 3 Department of Internal Medicine, Division of Infectious Diseases, Amsterdam University Medical Centers, location AMC, Amsterdam, The Netherlands; 4 Amsterdam Institute for Global Health and Development, Department of Global Health, Amsterdam University Medical Centers location AMC, Amsterdam, The Netherlands; 5 Department of Medical Microbiology and Infection Control, Amsterdam University Medical Centers location VUMc, Amsterdam, The Netherlands; 6 Present Address: Malawi Liverpool Wellcome Trust Clinical Research Programme, Blantyre, Malawi; 7 Present Address: Department of Public Health and Primary Care, Leiden University Medical Center, Leiden, The Netherlands; 8 Present Address: Julius Center for Health Sciences and Primary Care, University Medical Center Utrecht, Utrecht University, Universiteitsweg 100, Stratenum room 7.127, 3584 CG Utrecht, The Netherlands

**Keywords:** Microbiology, Diseases, Molecular medicine

## Abstract

Postmenopausal women and renal transplant recipients are at increased risk of recurrent urinary tract infections (RUTI). Urine and vaginal microbiota of premenopausal controls (N = 18) and RUTI cases (18), and of postmenopausal controls (30) and RUTI cases (20) with and without a renal transplant, were characterized using 16S rRNA sequencing. Participants did not have UTI symptoms at the time of sampling. Gram-negative uropathobionts (predominantly *Escherichia/Shigella*, *Pseudomonas, Klebsiella,* and *Acinetobacter*) had a much higher mean relative abundance in urine than vaginal samples, especially in premenopausal women. No statistically significant differences in mean relative abundances of bacterial groups were found within the premenopausal group or within the postmenopausal group by RUTI or renal transplant status without chronic antibiotic use. Comparing postmenopausal to premenopausal women, mean relative abundances of lactobacilli (especially *L. crispatus*) in urine and vaginal samples and of Gram-negative uropathobionts in urine were lower, and of BV-anaerobes and Gram-positive uropathobionts in urine and vaginal samples were higher. While RUTI in premenopausal women is predominantly caused by *Escherichia*, the causative organisms in postmenopausal women are likely more diverse. The relative importance of individual organisms is currently unknown. We recommend that future studies, including intervention studies, include longitudinal microbiota assessments.

## Introduction

The majority of women experience a urinary tract infection (UTI) at least once in their lifetime, and 20–30% of women with an uncomplicated UTI have a recurrence within six months^1^. Among ambulatory women without a urine catheter, the incidence rates of both UTIs and recurrent UTIs (RUTIs) are highest in postmenopausal women^1^. Renal transplant recipients are also at increased risk^2^; most of them are postmenopausal and all of them have to use immunosuppressive medication to prevent rejection of the renal transplant. UTIs are the most common infections following renal transplantation, and are associated with subsequent development of renal transplant rejection, impaired function, or loss^2–6^. Low-dose antibiotic treatment is commonly used to prevent UTI or RUTI in high risk ambulatory patients without a catheter, but is associated with bothersome side effects and development of antibiotic resistance^7^. Therefore, alternative prevention interventions are urgently needed, which requires a better understanding of the etiology of RUTI in this group.

Molecular characterization of the female urinary tract microbiome is gaining momentum^8–11^. Studies employing sterile collection procedures (suprapubic aspirate or transurethral catheterization) have shown that only about 50% of women have bacteria in the bladder^12^. When bacteria are present, women may or may not have any symptoms^1^. When symptoms are present, it is often not clear what causes them. This may not only depend on the bacterial taxa present, but also on the bacterial load, on expression of virulence factors, and on host immune system responses^11^. When no acute symptoms are present, long-term carriage of bacteria in the bladder may still cause complications such as urgency urinary incontinence^11,13^.

The vaginal microbiota of the majority of women is dominated by lactobacilli, most commonly *Lactobacillus crispatus* or *L. iners*^14^. Epidemiological studies suggest that lactobacilli-domination (with the exception of *L. iners*) protects women from bacterial vaginosis (BV; a condition in which the concentration of anaerobic bacteria in the vagina is increased) and from UTIs^15,16^. Postmenopausal women have much lower estrogen levels than premenopausal women, which may be one of the reasons why they have an increased risk of BV and UTIs^17^. Estrogen is required for glycogen to accumulate in vaginal epithelial cells. Lactobacilli metabolize vaginal glycogen into lactic acid, resulting in a low vaginal pH that keeps many other urogenital micro-organisms at bay^18^.

Ambulatory postmenopausal women, including renal transplant recipients, would benefit greatly from (R)UTI prevention interventions but have been understudied^16^. In this pilot study, we compared vaginal and urine bacterial microbiota compositions of pre- and postmenopausal women with and without RUTI and with and without a renal transplant, in order to generate hypotheses about which interventions might be tested in these patient populations in the future.

## Methods

### Study populations and data/sample collection procedures

We used data and samples from two studies in Amsterdam, the Netherlands: one in premenopausal and one in postmenopausal women. In both studies, RUTI cases were defined as women who self-reported three or more UTIs in the last year. Both studies were approved by ethics committees of the Amsterdam University Medical Centers, location AMC: the study in premenopausal women by the Medical Ethics Committee (protocol number 10/100; amendment 10/100#10.17.1729) and the study in postmenopausal women by the Biobank Ethics Committee (protocol number 2017_043#A201737). The research was performed in accordance with the Declaration of Helsinki. All participants provided written informed consent.

The premenopausal women had participated in a cross-sectional vaginal microbiota sub-study of the Healthy-Living-in-an-Urban-Setting (HELIUS) multi-ethnic cohort study in Amsterdam in 2015–2016^19,20^. Participants completed an online questionnaire prior to their clinic visit. They underwent a physical exam and self-collected two vaginal flocked swabs (stored dry) and a midstream urine sample (subsequently divided into 1 mL aliquots). All samples were immediately stored in a refrigerator at the clinic (for a maximum of 6 days) until transported to a − 20 °C storage facility. We selected all women who reported RUTI in the HELIUS vaginal microbiota study (n = 18 of 610) and matched them on age and ethnicity in a 1:1 ratio to women without RUTI (n = 18). The selected participants belonged to two different ethnic groups, which are known to have different microbiome compositions: Dutch descent (n = 26) or sub-Saharan African descent (n = 10)^19^.

The postmenopausal women provided samples for biobanking in the Amsterdam University Medical Centers, location AMC, in 2018. The biobank collection was established specifically to study RUTIs in AMC patients with renal diseases, and includes postmenopausal controls with or without RUTI. Women were seen by a study staff member during a clinic visit or at home. They completed a brief questionnaire, self-collected midstream urine, and donated one vaginal flocked swab (clinician-collected; stored dry). All samples were immediately stored in a refrigerator or cool box; vaginal swab heads and urine pellets were frozen at − 80 °C on the day of collection (Supplement-1). We selected 4 study groups: women who had not had a renal transplant without (n = 20) and with RUTI (n = 10) and renal transplant recipients without (n = 10) and with RUTI (n = 10). The latter group was split into two groups post-hoc: women who were (n = 4) or were not (n = 6) on prophylactic low-dose antibiotic therapy. Most women (48/50) were of Dutch descent. In the renal transplant recipient groups, we only included women more than 6 months after their renal transplantation. The following exclusion criteria were applied to all groups: current UTI defined by current antimicrobial use for this UTI, use of vaginal creams (including estrogen creams), vaginal washes or vaginal capsules in the past 30 days, antibiotic treatment within the past 30 days (with the exception of the low-dose treatments in renal transplant recipients as described above), and use of hormone replacement therapy.

### DNA extraction and sequencing

The samples of the pre- and postmenopausal women were processed separately (Supplement-1). Urine and vaginal samples were lysed using enzymes and mechanical bead-beating, followed by DNA extraction. The V3–V4 region of the 16S rRNA gene was sequenced on an Illumina MiSeq platform (Supplement-1).

### Microbiota data processing

Microbiota data processing was identical for data from the two studies. Sequence read pairs with perfect matching forward and reverse barcodes were assigned to their corresponding samples. Samples with fewer than 100 reads were removed from the datasets and we did not rarefy. The forward and reverse reads were length trimmed at 240 and 210 respectively, which were inferred and merged with amplicon sequence variants (ASVs) using DADA2 V.1.5.2^21^. Taxonomy assignment was done using the DADA2 implementation of the RDP classifier^22^ and the SILVA 16S reference database^23^.

We manually grouped ASVs into five bacterial groups, which were subdivided into 26 bacterial subgroups, based on the published literature^10,11,14^. A full listing of ASV allocations to bacterial groups and subgroups is provided in Supplement-2. The five bacterial groups were lactobacilli, BV-associated anaerobes, Gram-positive uropathobionts, Gram-negative uropathobionts, and other bacteria (any bacteria that did not fit into the previous groups). Lactobacilli were subdivided into *L. crispatus*, *L. iners*, *L. gasseri*, *L. jensenii*, and ‘other lactobacilli’; BV-anaerobes into *Gardnerella vaginalis*, *Atopobium vaginae*, *Prevotella* species, and ‘other BV-anaerobes’; Gram-positive uropathobionts into *Streptococcus agalactiae*, other Streptococci, *Actinotignum schaalii*, *Staphylococcus aureus, Aerococcus urinae*, *Enterococcus faecalis/faecium*, and ‘other Gram-positive uropathobionts’*;* Gram-negative uropathobionts into *Escherichia/Shigella*, *Klebsiella*, *Pseudomonas*, *Acinetobacter*, *Citrobacter*, *Enterobacter*, *Morganella*, *Serratia*, and ‘other Gram-negative uropathobionts’; and ‘other bacteria’ into *Bifidobacterium*, *Corynebacterium*, and ‘other bacteria’. For each urine and vaginal sample, relative abundances of all ASVs in a bacterial group or subgroup were added to arrive at the relative abundance of that group or subgroup in a particular sample. Alpha (Shannon and Chao1) and beta (Unifrac) diversities with bacterial subgroups as the unit of analysis were calculated using the phyloseq and microbiome package in R.

### Statistical analyses

Compositional differences between study groups of interest were determined by comparing mean alpha diversity measures using Wilcoxon rank-sum tests, and mean relative abundances of bacterial groups and subgroups using Wilcoxon rank-sum tests corrected for false discovery rate via Benjamin–Hochberg correction.

## Results

### Participant characteristics

The mean age of the premenopausal woman was 23.3 years (range 18–34) and of the postmenopausal women 64.1 years (range 53–77) (Table 1). A higher proportion of pre- than postmenopausal women were sexually active (most of whom were using hormonal contraception) and current smokers. Many postmenopausal women, including all renal transplant recipients, were chronic medication users. However, recent antibiotic use was rare in all groups, with the exception of the four renal transplant recipients with RUTI who were using low-dose antibiotics prophylactically. UTI symptoms in the past month (premenopausal women) or past year (postmenopausal women) were reported by 10.5–33.3% of women in the control groups, and 50.0–100% of women in the RUTI groups, but none of the women had UTI symptoms at the time of sampling. Current vaginal symptoms were reported by a higher proportion of RUTI cases than controls.

**Table 1:** Participant characteristics.

Variables n (%) or mean (range)	Premenopausal		Postmenopausal			
	Controls (N = 18)	RUTI (N = 18)	Controls (N = 20)	RUTI (N = 10)	RTR controls (N = 10)	RTR RUTI (N = 10)
Mean age in years	23.3 (18–34)	23.3 (18–34)	62.9 (53–75)	65.7 (56–73)	65.0 (57–76)	64.1 (55–77)
**Ethnicity**						
Dutch descent	13 (72.2)	13 (72.2)	20 (100)	9 (90.0)	10 (100)	9 (90.0)
SSA descent^1^	5 (27.8)	5 (27.8)	0	1 (10.0)	0	1 (10.0)
Body mass index	22.6 (17.9–31.7)	22.1 (18.0–30.2)	23.9 (17.3–36.1)	27.2 (22.0–38.3)	26.0 (20.9–35.6)	24.0 (15.4–33.2)
Sexually active past 6 months	13 (72.2)	14 (77.8)	10 (52.6)	1 (10.0)	4 (40.0)	3 (30.0)
If sexually active, condom use always (vs. inconsistent or never)	1 (7.7)	2 (14.3)	ND	ND	ND	ND
Sex in past 48 h	ND	ND	0	0	0	0
Has at least one child	2 (11.1)	4 (22.2)	ND	ND	ND	ND
Using hormonal contraception^2^	9 (50.0)	11 (61.1)	NA	NA	NA^2^	NA^2^
Currently breastfeeding	0	0	NA	NA	NA	NA
Current smoker	7 (38.9)	8 (44.4)	2 (10.0)	1 (10.0)	0	0
Current chronic medication use^3^	ND	ND	8 (42.1)	7 (70.0)	10 (100)	10 (100)
Recent antibiotic use^4^	1 (5.6)	2 (11.1)	0	0	0	4 (40.0)
Antibiotic use in past 6 months^5^	ND	ND	0	8 (80.0)	4 (40.0)	7 (70.0)
Diabetes mellitis diagnosis	0	0	0	0	3 (30.0)	4 (40.0)
UTI symptoms in past month	6 (33.3)	9 (50.0)	ND	ND	ND	ND
UTI symptoms in last 12 months^6^	ND	ND	2 (10.5)	10 (100)	2 (20.0)	10 (100)
Vaginal symptoms in past month	3 (16.7)	12 (66.7)	ND	ND	ND	ND
Sometimes has vaginal yeast infection(s)	ND	ND	3 (15.8)	2 (20.0)	1 (10.0)	4 (40.0)
Sometimes has other vaginal symptom(s)	ND	ND	0	4 (40.0)	2 (20.0)	5 (50.0)

Abbreviations: *NA* not applicable, *ND* not done/not asked, *RTR* renal transplant recipients, *RUTI* recurrent urinary tract infection (defined as at least three UTIs in the last year), *SSA* sub-Saharan Africa.

^1^Among premenopausal women, includes 4 women of Afro-Surinamese descent and one woman of Ghanaian descent in each group.

^2^75% of premenopausal hormonal contraceptive users were using a combined oral contraceptive pill and the other 25% Nuvaring or an intrauterine device. One woman in each of the indicated postmenopausal groups was using a vaginal estradiol cream.

^3^Does not include antibiotics. Includes medications for blood pressure and/or cholesterol lowering, and calcium/vitamin D, in all groups. Additional medications among non-RTR women were thyroid hormone (one in the control and one in the RUTI group), obstructive pulmonary disease inhalation therapy (one control and one RUTI) and betahistine (RUTI). Additional medications among RTR women included immunosuppressive, phosphate-binding, and diuretic medications, erythropoietin mimetics, and metformin (one woman also used insulin; RTR women used 4–14 medications per person).

^4^In the last 14 days in the premenopausal group and the last 30 days in the postmenopausal group. Includes doxycycline and two unknown antibiotics in the control group, and fosfomycin (prophylactic use), nitrofurantoin (prophylactic use), and ertapenem (recent treatment course on top of nitrofurantoin prophylaxis) in the RTR RUTI group.

^5^Includes fosfomycin, nitrofurantoin, ciprofloxacin, trimethoprim, amoxicillin/amoxicillin with clavulanic acid, and unknown antibiotics.

^6^The women in the control groups reported to have had UTI symptoms only once in the last 12 months whereas the women in the RUTI groups reported 3–12 episodes with three women reporting to have symptoms permanently.

### Microbiota overviews and diversities

**Figure 1:**
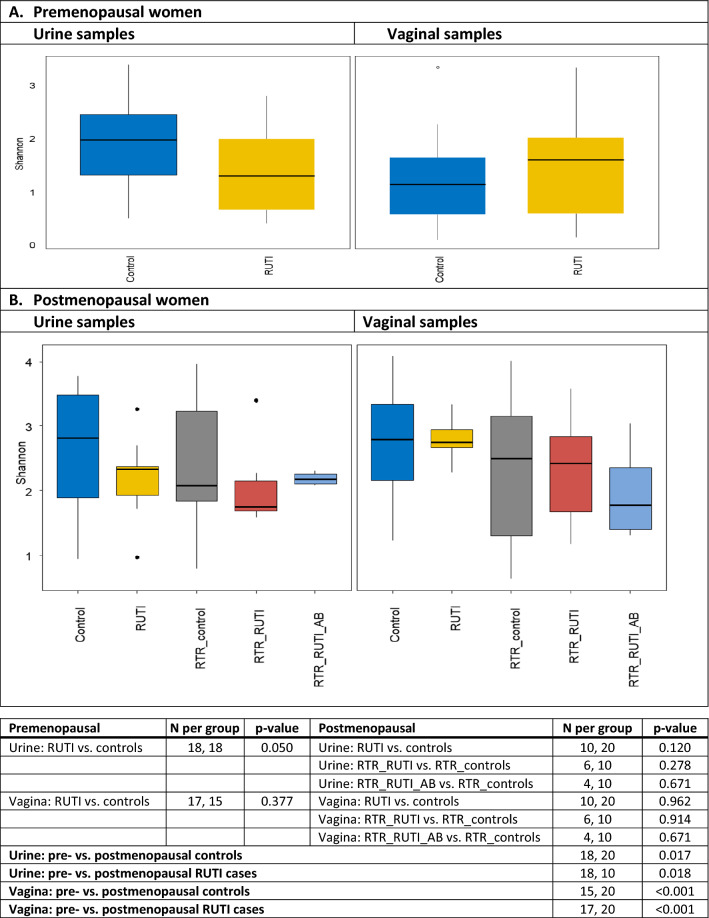
Shannon alpha diversity of urine and vaginal samples by study group. Abbreviations: *AB* on antibiotic prophylaxis, *RTR* renal transplant recipient, *RUTI* recurrent urinary tract infection (defined as at least three UTIs in the past year). The figure shows the mean Shannon alpha diversity index for each group with 95% confidence intervals. *p* Values are by Wilcoxon rank-sum tests. Data for Chao1 indexes and richness values are shown in Supplement-1.

Heatmaps depicting relative abundances of all 26 bacterial subgroups in individual samples by study group are shown in Supplement-1 Figures S1A (urine samples) and S1B (vaginal samples). The Shannon alpha diversities of urine and vaginal samples were within the same ranges (Fig. 1). Both the urine and vaginal mean Shannon alpha diversities were significantly lower in premenopausal than in postmenopausal controls (*p* = 0.017 and < 0.001, respectively) and RUTI cases (*p* = 0.018 and *p* < 0.001, respectively). Results were similar for Chao1 alpha diversity and richness (Supplement-1: Figure S2). The mean Shannon alpha diversity of urine samples from premenopausal RUTI cases was lower than for premenopausal controls (*p* = 0.050), but this was not the case for Chao1 and richness measures, nor for any of the diversity measures of vaginal samples (Shannon indexes in Fig. 1, Chao1 and richness measures in Supplement-1: Figure S2). None of the mean diversity measures of urine or vaginal samples differed in the different groups of postmenopausal women (Shannon indexes in Fig. 1, Chao1 and richness measures in Supplement-1: Figure S2). The weighted and unweighted UniFrac distances of urine and vaginal samples from the different study groups completely overlapped, suggesting no significant differences in beta diversities either (Supplement-1: Figure S3).

### Microbiota compositions in premenopausal women

**Figure 2:**
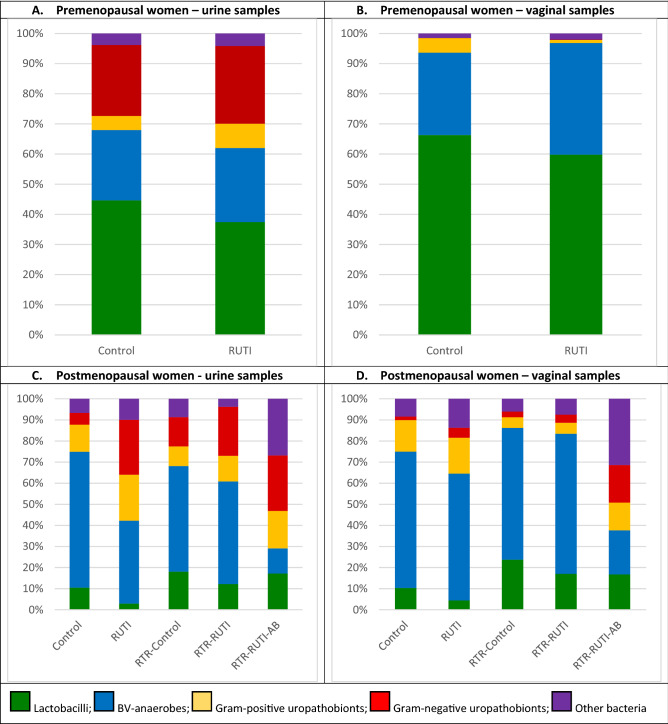
Mean relative abundances of bacterial groups in urine and vaginal samples by study group. Abbreviations: *AB* on antibiotic prophylaxis, *BV* bacterial vaginosis, *RTR* renal transplant recipient, *RUTI* recurrent urinary tract infection (defined as at least three UTIs in the past year). Sample sizes: premenopausal urine controls (18) and RUTI (18); premenopausal vaginal samples controls (15) and RUTI (17); postmenopausal controls (20), RUTI (10), RTR controls (10), RTR RUTI (6), and RTR using antibiotic prophylaxis (4) for both urine and vaginal samples.

**Figure 3:**
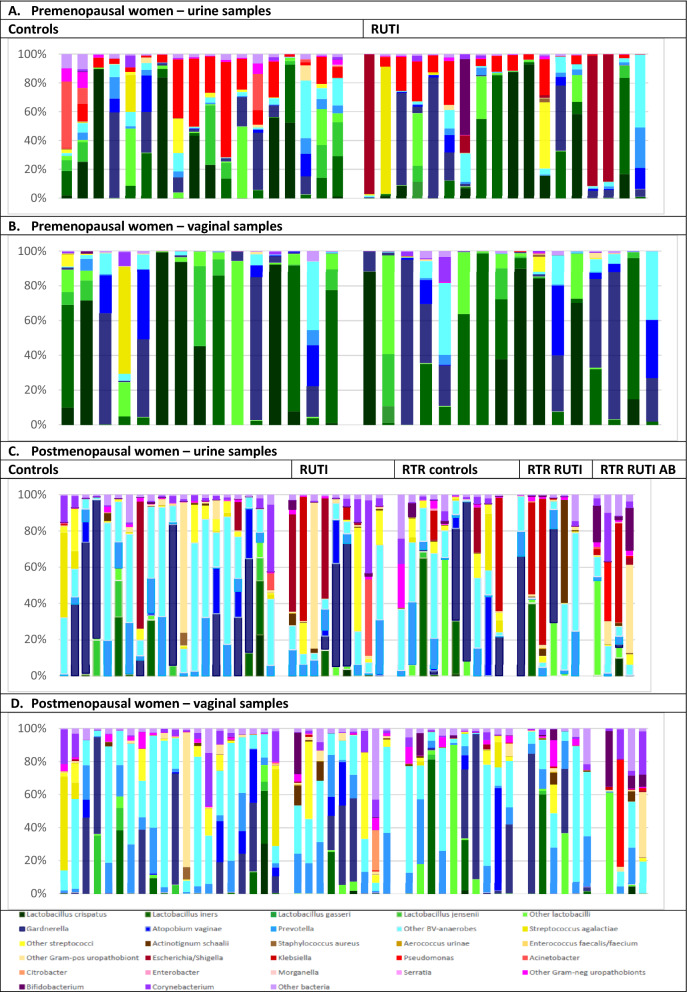
Relative abundances of bacterial subgroups in urine and vaginal samples by study group. Abbreviations: *AB* on antibiotic prophylaxis, *BV* bacterial vaginosis, *RTR* renal transplant recipient, *RUTI* recurrent urinary tract infection (defined as at least three UTIs in the past year). Each bar represents one unique sample. Sample sizes: premenopausal urine controls (18) and RUTI (18); premenopausal vaginal samples controls (15) and RUTI (17); postmenopausal controls (20), RUTI (10), RTR controls (10), RTR RUTI (6), and RTR using antibiotic prophylaxis (4) for both urine and vaginal samples.

The urine and vaginal microbiota of premenopausal controls consisted mostly of lactobacilli and BV-anaerobes, with low mean relative abundances of Gram-positive uropathobionts and ‘other bacteria’, and varying mean relative abundances of Gram-negative uropathobionts (Figs. 2A,B, Supplement-1: Tables S1, S2 and Figure S4A,B). The most common bacterial subgroups were (percentages indicate the mean relative abundance range when including all samples from all premenopausal women)*: L. crispatus* (16.0–28.1%), *L. iners* (9.4–23.7%), *G. vaginalis* (10.3–22.6%), *Streptococcus* (0.6–7.6%), *L. jensenii* (1.2–6.8%), *A. vaginae* (2.1–6.3%), and *Prevotella* (0.6–3.2%; Fig. 3A,B, Supplement-1: Tables S1, S2). ‘Other lactobacilli’ (mostly consisting of lactobacilli that could not be identified at species level; 4.9–10.9%) and ‘other BV-anaerobes’ (5.1–8.7%) were also common but *L. gasseri* and ‘other Gram-positive uropathobionts’ were rare. Premenopausal RUTI cases compared to controls had higher mean relative abundances of BV-anaerobes, and lower mean relative abundances of lactobacilli, in both urine and vaginal samples, but these differences did not reach statistical significance (Supplement-1: Tables S1, S2). Mean relative abundances of Gram-positive uropathobionts were similarly low across sample types and groups (Supplement-1: Tables S1, S2).

The largest sample type and group differences were seen for Gram-negative uropathobionts: the mean relative abundances were 25.8% and 23.5% (*p* = 0.828) in urine samples of RUTI cases and controls, respectively, but negligible in vaginal samples of both groups. *Escherichia/Shigella* (16.1% in RUTI cases and 0% in controls; *p* = 0.612) and *Pseudomonas* (9.2% in RUTI cases and 16.6% in controls; *p* = 0.512) were the most common Gram-negative uropathobionts in urine samples.

Premenopausal RUTI cases and controls were matched on ethnicity. Figure S5 confirms that this matching was needed. Women of sub-Saharan African descent had a lower mean relative abundance of lactobacilli than women of Dutch descent in both urine (28.4 and 46.6%, respectively) and vagina (36.8% and 75.5%, respectively), a higher mean relative abundance of BV-anaerobes in both urine (37.4% and 17.4%, respectively) and vagina (52.2% and 22.7%, respectively); but similar mean relative abundances of Gram-positive and Gram-negative uropathobionts (Supplement-1: Figure S5).

### Microbiota compositions in postmenopausal women

The urine and vaginal microbiota of postmenopausal controls without RUTI or renal transplant consisted of mixtures of lactobacilli, BV-anaerobes, Gram-positive and Gram-negative uropathobionts, and ‘other bacteria’ (Fig. 2C,D, Supplement-1: Tables S1, S2 and Figure S4C,D). The most common bacterial subgroups were (percentages indicate the mean relative abundance range when including all samples from all postmenopausal women except the four women who used antibiotics prophylactically): ‘other BV-anaerobes’ (4.3–30.6%), *G. vaginalis* (13.3–21.0%), *Prevotella* (7.9–18.4%), *Streptococcus* (0.8–12.1%), ‘other lactobacilli’ (0.9–12.0%), *L. iners* (1.7–11.2%), ‘other Gram-positive uropathobionts’ (0.2–9.8%), and *A. vaginae* (0–7.2%; Fig. 3C,D, Supplement-1: Tables S1, S2). *L. crispatus* and *L. jensenii* were rare. Gram-negative uropathobionts were again present in urine samples but not vaginal samples, predominantly *Escherichia/Shigella* (0–12.2%), *Klebsiella* (0–8.3%)*,* and *Acinetobacter* (0–4.6%).

Postmenopausal RUTI cases compared to controls had non-significantly higher mean relative abundances of Gram-positive and Gram-negative uropathobionts, and lower mean relative abundances of lactobacilli and BV-anaerobes, in urine (but not vaginal) samples. Renal transplant recipients resembled other postmenopausal women, with the exception of prophylactic antibiotic users. The latter group had a very low mean relative abundance of lactobacilli in both urine and vaginal samples, while the other 4 bacterial groups were overrepresented (Figs. 2C,D, 3C,D, Supplement-1: Tables S1, S2).

### Microbiota comparisons between pre- and postmenopausal women

Comparing postmenopausal to premenopausal controls, the mean relative abundances of lactobacilli were significantly lower in both urine samples (10.5% vs. 44.7%; *p* < 0.001) and vaginal samples (10.3% vs. 66.3%; *p* < 0.001). The mean relative abundances of all other bacterial groups were significantly higher in postmenopausal controls with one exception: the mean relative abundance of Gram-negative uropathobionts in urine samples was lower (5.6% vs. 23.5%; *p* < 0.001) and negligible in vaginal samples of both groups.

**Figure 4:**
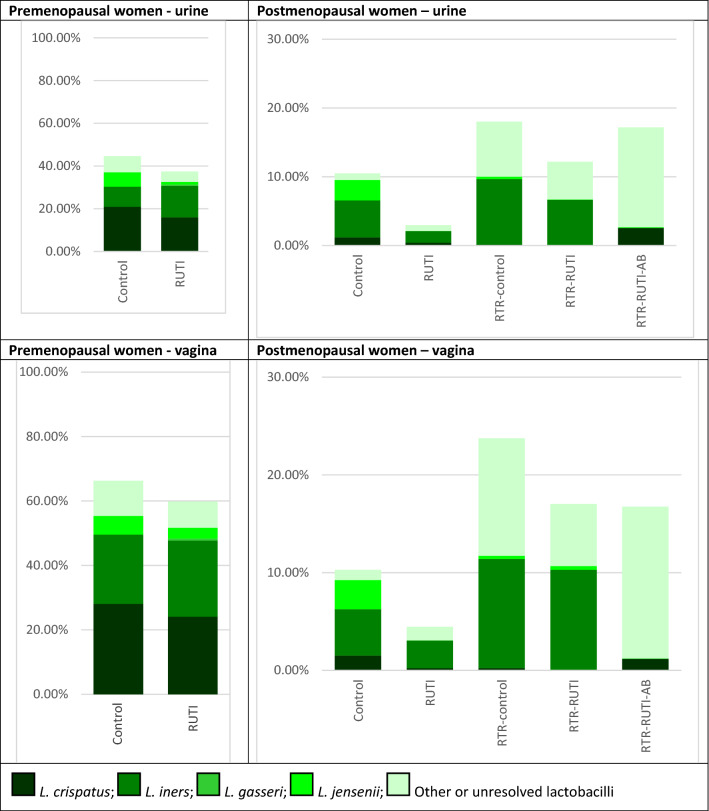
Mean relative abundances of *Lactobacillus* species by urine and vaginal samples by study group. Abbreviations: *AB* on antibiotic prophylaxis, *RTR* renal transplant recipient, *RUTI* recurrent urinary tract infection (defined as at least three UTIs in the past year). Unresolved lactobacilli could only be identified at genus level, not at species level. Sample sizes: premenopausal urine controls (18) and RUTI (18); premenopausal vaginal samples controls (15) and RUTI (17); postmenopausal controls (20), RUTI (10), RTR controls (10), RTR RUTI (6), and RTR using antibiotic prophylaxis (4) for both urine and vaginal samples.

The most common *Lactobacillus* species in urine and vaginal samples of premenopausal controls was *L. crispatus* (20.9% and 28.1%, respectively) and in postmenopausal controls *L. iners* (5.4% and 4.8%, respectively; Fig. 4). Samples of premenopausal RUTI cases contained similar mean relative abundances of *L. crispatus* (16.0% in urine and 24.1% in vaginal samples) and *L. iners* (14.8% and 23.7%, respectively), whereas those of postmenopausal RUTI cases contained very low mean relative abundances of these lactobacilli (all below 3%; Supplement-1: Tables S1, S2). Individual variation in lactobacilli composition was large in both premenopausal and postmenopausal women across study groups (Supplement-1: Figure S5).

While the mean relative abundances of *G. vaginalis* and *A. vaginae* were similar across study groups except prophylactic antibiotic users, the mean relative abundance of *Prevotella* and ‘other BV-anaerobes’ were significantly higher in post- than premenopausal controls and RUTI cases (all comparisons *p* < 0.05; Supplement-1: Tables S1, S2). Streptococci were the most common Gram-positive uropathobionts across sample types and groups, but were significantly more common in urine and vaginal samples of post- than premenopausal controls and RUTI cases (all comparisons *p* < 0.05; Supplement-1: Tables S1, S2). This was also the case for the much less relatively abundant Gram-positive uropathobionts *Actinotignum schaalii* and *Aerococcus urinae* and the Gram-negative uropathobionts *Klebsiella* and *Pseudomonas*. The mean relative abundances of *Escherichia/Shigella* in urine samples were not significantly different between post- and premenopausal controls (*p* = 0.737) or RUTI cases (*p* = 0.979).

## Discussion

The women in our study did not have UTI symptoms at the time of sampling, but the RUTI cases had reported at least three UTI episodes in the past year. We hypothesized that the organisms causing these RUTIs have a long-term presence in these women’s urinary and/or vaginal microbiota, and that we would therefore see differences in microbiota compositions even in the absence of UTI symptoms. The urine and vaginal microbiota compositions of pre- and postmenopausal women indeed differed significantly, but we did not find any significant microbiota differences by RUTI or renal transplant status in the absence of chronic antibiotic use. We had limited statistical power, which might explain why some observed differences did not reach statistical significance. However, we were able to show many statistically significant differences by menopausal status despite the modest sample size, and menopausal status may therefore be a stronger determinant of both urine and vaginal microbiota composition than RUTI or renal transplant status. Other groups have shown similar differences between pre- and postmenopausal women for urine^24,25^ and vaginal samples^26,27^.

We expected overlap between the bacterial taxa in the urinary and genital tracts given their close anatomical proximity to one another and to the rectum, as has been found by others^28^. We also expected midstream urine samples collected by women themselves to contain organisms that originated from the vulva or perineum instead of the urinary tract. Lactobacilli, BV-anaerobes, and Gram-positive uropathobionts were indeed identified in urine and vaginal samples from most women, and we cannot be certain of exact origins. However, we also observed some strong niche effects. Lactobacilli dominated vaginal samples to a larger extent than urine samples, and this niche effect was stronger in premenopausal than in postmenopausal women. We also saw differences in the most relatively abundant *Lactobacillus* species: *L. crispatus* in premenopausal women and *L. iners* in postmenopausal women. In contrast, Gram-negative uropathobionts were almost exclusively found in urine: the mean relative abundance was highest in urine from premenopausal women and almost absent from vaginal samples of these same women; a similar trend was seen for postmenopausal women but less pronounced.

In clinical practice, UTI diagnostic testing and empirical treatments assume that Gram-negative uropathobionts, and especially *Escherichia coli*, are the most common causative organisms^29^. While our microbiota data from RUTI cases suggest that this is likely true for premenopausal women, they also suggest that the causative organisms in postmenopausal women may be much more diverse. The potential role of Gram-positive uropathobionts has been highlighted by others^10,11^, and some *Streptococcus*, *Staphylococcus*, and *Enterococcus* species, among others, are increasingly recognized as causing UTIs. In contrast, potential roles for some of the most pathogenic BV-anaerobes (such as *G. vaginalis*), and perhaps also vaginal yeasts, have been considered much less^10^. The potential role of BV-anaerobes is difficult to study because they are very commonly present in both urine and vaginal samples from diverse groups of women. An interesting study in mice showed that bladder exposure to *G. vaginalis* triggered *E. coli* egress from latent bladder reservoirs, suggesting that interactions between uropathobionts and BV-anaerobes may be important in UTI pathogenesis and not the mere presence of BV-anaerobes alone^30^.

Our study had limitations. We already mentioned the limited statistical power, which may have been exacerbated by large inter-individual variation in microbiota compositions; we tried to mitigate this by carefully selecting our study groups. The study was cross-sectional, not quantitative (i.e. data are relative abundances and not bacterial loads), and Illumina MiSeq sequencing does not provide strain-level or functional insights. We therefore missed microbiota composition, bacterial load, and functional changes over time, including changes that are expected to take place during acute UTI episodes. Importantly, urine sample collection and urine and vaginal swab processing procedures differed between the two studies (see Supplement-1 for details). However, samples within each study were collected and processed in an identical manner, making all within-study comparisons valid. Comparisons between the two studies should be interpreted with caution due to some of the above-mentioned procedural differences. However, mock community controls in each study (which included *Lactobacillus*, Gram-positive uropathobionts, and Gram-negative uropathobionts) provided accurate results, and we used the same bioinformatics pipeline in each study.

## Conclusions

Our data suggest that postmenopausal status may be a more important determinant of microbiota composition that RUTI or renal transplant status. While RUTI in premenopausal women is predominantly caused by *Escherichia/Shigella*, the causative organisms in postmenopausal women are likely more diverse. An improved understanding of the organisms involved in RUTI could lead to improved diagnostics and targeted treatments, which in turn may reduce the development of antimicrobial resistance caused by suboptimal empirical treatments. This requires longitudinal studies capturing women before, during, and after (R)UTI episodes, and incorporating sequencing as well as quantification of organism loads. We highly recommend that all future intervention studies also incorporate longitudinal microbiota assessments to gain better insight into the effects of these interventions on microbiota compositions and organism loads.

## Supplementary Information

Supplementary Information 1.

Supplementary Information 2.

## Data Availability

The HELIUS data are owned by the Amsterdam University Medical Centers, location AMC, in Amsterdam, The Netherlands. Any researcher can request the data by submitting a proposal to the HELIUS Executive Board as outlined at http://www.heliusstudy.nl/en/researchers/collaboration. The HELIUS Executive Board will check proposals for compatibility with the general objectives, ethical approvals and informed consent forms of the HELIUS study. There are no other restrictions to obtaining the data and all data requests will be processed in the same manner.
