# Supplementary material for: Urine and vaginal microbiota compositions of postmenopausal and premenopausal women differ regardless of recurrent urinary tract infection and renal transplant status
Source: Sci Rep. 2022 Feb 17;12:2698. doi: 10.1038/s41598-022-06646-1 (PMC8854725; doi:10.1038/s41598-022-06646-1)
Supplement: Supplementary file 1 — Supplementary Information 1. [file 41598_2022_6646_MOESM1_ESM.docx]

**SUPPLEMENT 1**

**Urine and vaginal microbiota compositions of postmenopausal and premenopausal women differ regardless of recurrent urinary tract infection and renal transplant status**

**Authors:** Floor Hugenholtz, Charlotte van der Veer, Matty Terpstra, Hanneke Borgdorff, Robin van Houdt, Sylvia Bruisten, Suzanne Geerlings, Janneke van de Wijgert

**Supplementary methods**

*Sample collection, processing and sequencing - premenopausal women (HELIUS study)*

Premenopausal HELIUS study participants self-collected two vaginal swabs (FLOQSwabs, Copan Diagnostics Inc., Murrieta, CA, USA) and a midstream urine sample during a clinic visit. The two vaginal swab heads were stored dry in one tube. The urine sample was stored in 1 ml aliquots. All samples were immediately stored in a refrigerator at the clinic (for a maximum of 6 days) until transported to a -20°C storage facility.

At the time of DNA extraction, urine aliquots were centrifuged and the pellets were resuspended in 400 µl urine. The resuspended urine and the vaginal swab heads were lysed with lysozyme (10mg/mL; Sigma Aldrich, St. Louis, MO, USA), mutanolysin (>4U/µL; Sigma Aldrich, St. Louis, MO, USA), and lysostaphin (1mg/mL; AMBI, New York, NY, USA). Lysed samples were further treated using proteinase K (20mg/mL; Thermo Fisher, Waltham, MA, USA) and RNase A (10mg/mL; Thermo Fisher, Waltham, MA, USA). A mechanical bead-beating step followed: 5 m/s for 40 seconds (MagnaPure, Roche Diagnostics, and Basel, Switzerland). DNA was extracted from the crude lysates using a ChemaGen extraction robot (PerkinElmer, Baesweiler, Germany).

Dual indexed universal primers (319F and 806R) were used for PCR amplification of the V3-V4 regions of the 16S rRNA genes as described by Fadrosh (1) with minor modifications (no diverse length linker sequences were used). DNA was amplified in a 25 μl reaction volume using 1.25 μl of a 10 µM concentration of 319F 5’-ACTCCTACGGGAGGCAGCAG-3’ forward primer and 1.25 μl of a 10 µM concentration of 806R 5’-GGACTACHVGGGTWTCTAAT-3’ reverse primer, 12.5 μl NEB Next HF 2x PCR Master Mix (New England Biolabs, Hitchin, UK), 9 μl of nuclease-free water and 1 μl of DNA extraction product. The amplification program was first denaturation at 98°C for 30s, 10 cycles consisting of a denaturation cycle at 98°C of 10s, an annealing cycle at 58°C for 30s, an extension cycle at 72°C for 30s, and a final extension cycle at 72°C of 5 min. The second PCR round was to barcode V3-V4 sequences by a dual-index approach using standard Illumina Nextera XT index kit v2 (Illumina, San Diego, CA, USA). The barcoding was performed in a 25 μl reaction volume using 2.5 μl of Index 1 primer, 2.5 μl of Index 2 primer, 12.5 μl NEB Next HF 2x PCR Master Mix and 7.5 μl sample. The first denaturation cycle took 3 min at 98°C and was followed by 15 cycles consisting of a denaturation cycle at 98°C of 30s, an annealing cycle at 55°C of 30s, an extension cycle at 72°C of 30s, and a final extension cycle at 72°C of 5 min.

PCR products were pooled and normalized, and purified with Agencourt AMPure XP magnetic beads (BeckmanCoulter, Fullerton, CA, USA). Paired-end sequencing (2x300bp) was performed on an Illumina MiSeq instrument (Illumina, San Diego, CA, USA).

*Sample collection, processing and sequencing - postmenopausal women (AMC kidney disease biobank)*

Postmenopausal women were seen by a study staff member during a clinic visit or at home. They completed a brief questionnaire, self-collected midstream urine, and donated one vaginal flocked swab (clinician-collected; FLOQSwabs, Copan Diagnostics Inc., Murrieta, CA, USA). Vaginal swabs were stored in dry tubes and were kept in a cold box, after which they were stored as soon as possible but always within one hour at -80 °C until further processing. Urine samples were also initially kept in a cool box, after which they were placed in a refrigerator until further processing that same day. Urine samples were centrifuged at 1550g, after which the pellet was stored at -80°C in two 2 ml cryovials per participant. If the tube contained little pellet, it was topped off with extra fresh urine until a volume of about 2 ml.

Just prior to DNA extraction, the stored 2 ml urine and vaginal samples were vortexed and 1 ml each was used for DNA extraction. DNA was extracted using a combination of repeated bead-beating (2) and the Maxwell RSC Blood DNA kit (Promega, Leiden, Netherlands) with STAR (Stool Transport and Recovery) buffer (Roche, Basel, Switzerland). Following repeated bead-beating, heating and centrifugation, 250 µl supernatant was used with the Maxwell RSC Blood DNA kit, and the DNA was eluted in 50 µl Nuclease free water.

Twenty nanograms of DNA was used for amplification of the V3-V4 regions of the 16S rRNA genes using the 341F forward and 805R reverse primers. The PCR was performed in a total volume of 30 µl containing 1× HF buffer (Thermo Fisher Scientific, Waltham, MA, USA ), 200 µM dNTP Mix (10 mM; Promega, Leiden, the Netherlands), 1 U of Phusion Green High-Fidelity DNA Polymerase, 500nM of the forward 8-nt sample-specific barcode primer 341F (5’- CCTACGGGNGGCWGCAG-3’), 500nM of reverse 8-nt sample-specific barcode primer 805R (5’ GACTACHVGGGTATCTAATCC-3’), 20 ng of template DNA, and nuclease free water. No diverse length linker sequences were used. The amplification program was initial denaturation at 98°C for 30s; 25 cycles of denaturation at 98°C for 10s, annealing at 55°C for 20s, elongation at 72°C for 90s; and an extension at 72°C for 10 min (3). The size of the PCR products (~540 bp) was confirmed by gel electrophoresis using 4 µl of the amplification reaction mixture on a 1% (w/v) agarose gel containing ethidium bromide (AppliChem, Darmstadt, Germany).

PCR products were purified with Agencourt AMPure XP magnetic beads (BeckmanCoulter, Fullerton, CA, USA) according to manufacturer’s instructions using 96 wells format with Biomex FX (Beckman Coulter, Brea, CA, USA) and 60 µl Nuclease Free Water (Qiagen, Hilden, Germany). Purified PCR product was quantified by the use of Qubit dsDNA BR Assay Kit in combination with FLUOstar OPTIMA (BMG LaBTECH, Ortenberg, Germany). The PCR purified product was equimolar mixed and loaded on the Illumina MiSeq with the MiSeq V3 - 600 cycle kit, as instructed by Illumina.

*Comparison of procedures in the two studies:*

|  | **Premenopausal women** | **Postmenopausal women** |
| --- | --- | --- |
| Urine sample collection | Midstream self-collected | Midstream self-collected |
| Urine sample storage and processing | 1 ml aliquots at -20°C; centrifuged and resuspended in 400 μl urine just prior to DNA extraction. | Centrifuged after collection; pellet topped up to 2 ml, stored at -80°C. Vortexed, and 1 ml used for DNA extraction. |
| Vaginal sample collection | Flocked self-collected swabs | Flocked self-collected swabs |
| Vaginal sample storage | Swab heads stored dry at -20°C | Swab heads stored dry at -80°C |
| DNA extraction, all samples | Lysozyme, mutanolysin, lysostaphin, proteinase K, RNAse, bead-beating. | Maxwell RSC Blood DNA kit (incl. proteinase K) and repeated bead-beating. |
| V3-V4 amplification | Primers 319F, 806R; 1μl DNA extract in 25 μl reaction volume; dual index barcoding without diverse length linker sequences | Primers 341F, 805R; 20 ng DNA extract in 30 μl reaction volume; dual index barcoding without diverse length linker sequences |
| Sequencing | Illumina MiSeq, 2x300bp | Illumina MiSeq, V3-600 cycle |
| Bioinformatics pipeline | Identical between the two studies | Identical between the two studies. |

The two studies handled urine samples differently but vaginal samples were handled in an identical manner. The DNA extraction protocols were not identical but both protocols contained enzymatic lysis, proteinase K treatment, and bead-beating. The PCR protocols both targeted the V3-V4 region of the 16S rRNA gene albeit using different primers. Both studies used the Illumina MiSeq platform and MiSeq protocols generating reads with an average length of 500-600bp (which is needed to be able to differentiate between *Lactobacillus* species). We used the exact same bioinformatics pipeline in each study. Samples within each study were handled in an identical manner and within-study comparisons are therefore valid. Comparisons between the two studies (between pre- and postmenopausal women) should be interpreted with caution. However, we sequenced mock community controls in each study (which included *Lactobacillus*), which provided accurate results. We therefore believe that exploratory comparisons can be done.

**Supplementary results**

Not including the 4 samples with fewer than 100 reads, the mean number of reads was 16,546 (range 231-62,260) for premenopausal urine samples (N=36), 38,261 (5,557-99,526) for premenopausal vaginal samples (N=32), 31,603 (2,078-55,782) for postmenopausal urine samples (N=50), and 29,252 (2,031-29,769) for postmenopausal vaginal samples (N=50).

Some Gram-negative uropathobionts (*Escherichia/Shigella*, *Klebsiella*, *Pseudomonas*) could reach mean relative abundances above 15% in urine samples while being absent in vaginal samples. Almost all premenopausal women had Gram-negative uropathobionts in their urine samples, with 7/18 RUTI cases and 9/18 controls having a relative abundance of more than 20%. Only two women in each group had >20% Gram-positive uropathobionts. While >20% Gram-negative uropathobionts was also common in urine samples of postmenopausal women with RUTI (3/9 cases), they were just as likely to have >20% Gram-positive uropathobionts in their urine (also 3/9 cases).

**References**

1. Fadrosh DW, Ma B, Gajer P, Sengamalay N, Ott S, Brotman RM, et al. An improved dual-indexing approach for multiplexed 16S rRNA gene sequencing on the Illumina MiSeq platform. Microbiome. 2014 Dec;2(1):6.

2. Yu Z, Morrison M. Improved extraction of PCR-quality community DNA from digesta and fecal samples. BioTechniques. 2004 May;36(5):808–12.

3. Kozich JJ, Westcott SL, Baxter NT, Highlander SK, Schloss PD. Development of a Dual-Index Sequencing Strategy and Curation Pipeline for Analyzing Amplicon Sequence Data on the MiSeq Illumina Sequencing Platform. Appl Environ Microbiol. 2013 Sep 1;79(17):5112–20.

**Figure S1: Heatmaps by sample type and study groups**

| 1. **Urine samples** |
| --- |
| **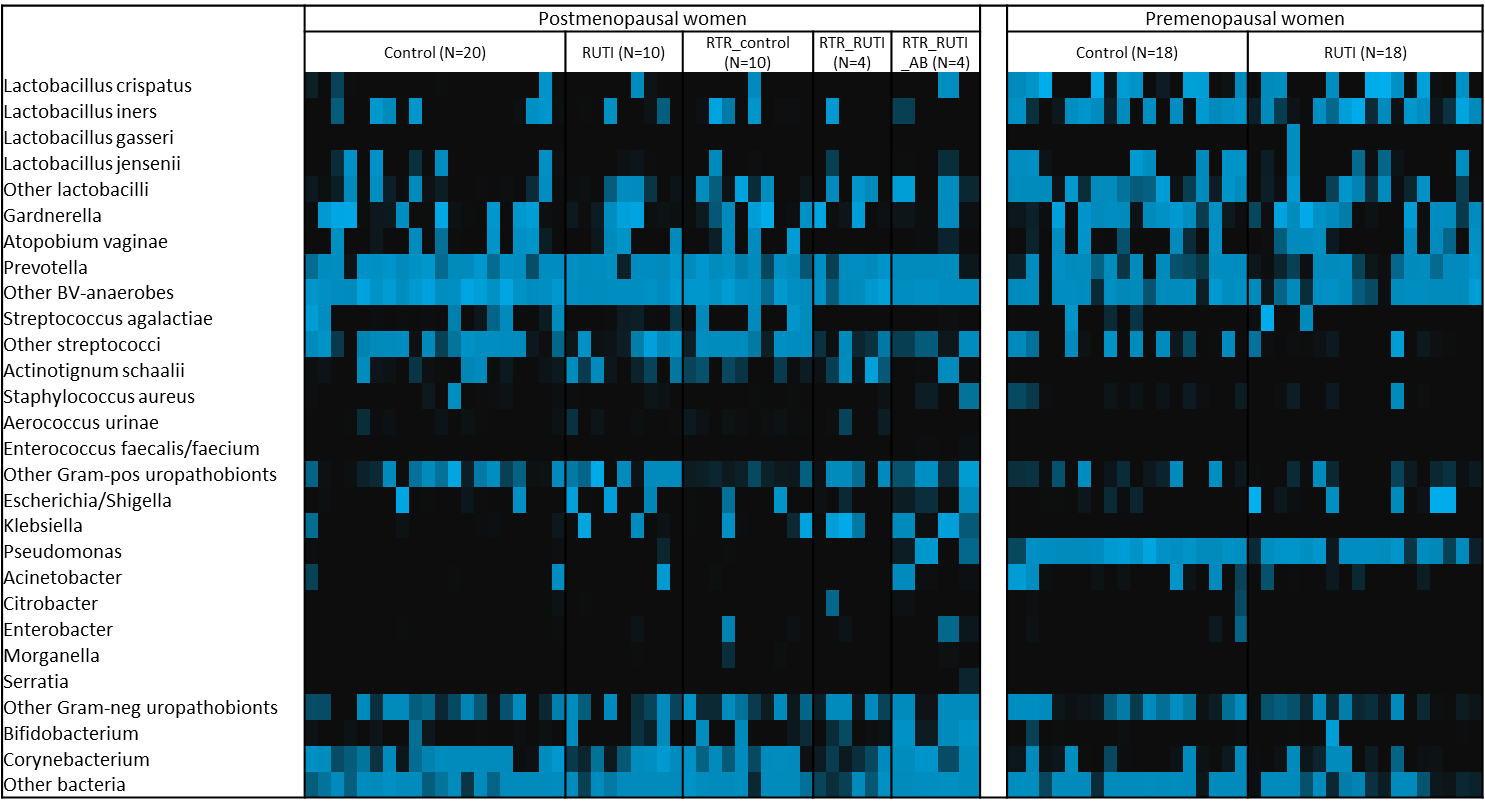** |

Abbreviations: AB=on antibiotic prophylaxis; RTR=renal transplant recipient; RUTI=recurrent urinary tract infection (defined as at least three UTIs in the past year).

| 1. **Vaginal samples** |
| --- |
| **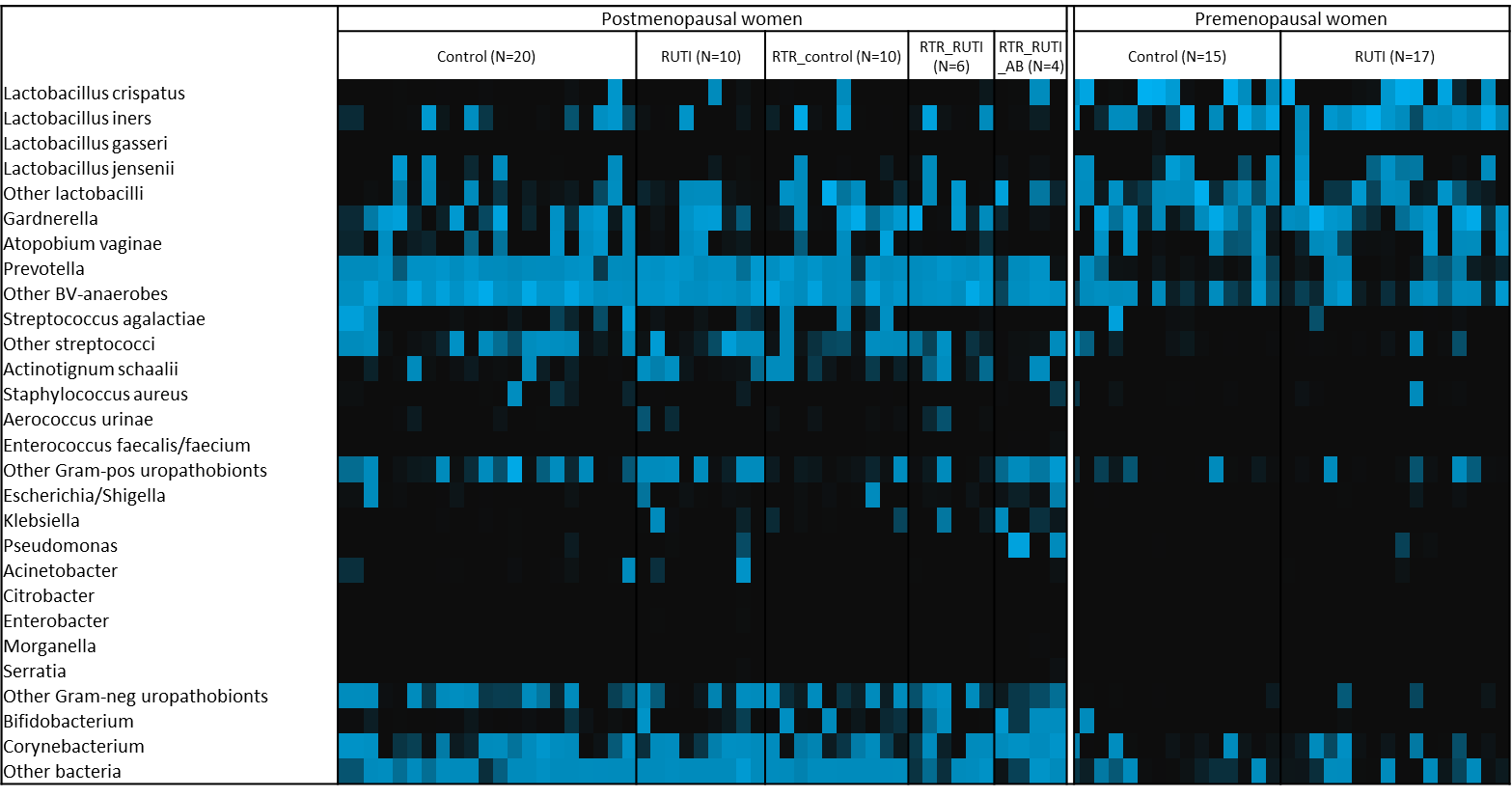** |

Abbreviations: AB=on antibiotic prophylaxis; RTR=renal transplant recipient; RUTI=recurrent urinary tract infection (defined as at least three UTIs in the past year). Each column represents one unique sample.

**Figure S2A: Chao 1 alpha diversity and richness by type of sample and study group**

| **Premenopausal women: Chao 1 indexes** | **Premenopausal women: richness** |
| --- | --- |
| Urine  Vaginal  Control  RUTI  Control  RUTI  100  200 | Control  RUTI  Control  RUTI  50  100  150  200  Urine  Vaginal |
| **Postmenopausal women: Chao 1 indexes** | **Postmenopausal women: richness** |
| Control  RUTI  RTR_control  RTR_RUTI  RTR_RUTI_AB  Control  RUTI  RTR_control  RTR_RUTI  RTR_RUTI_AB  200  400  600  Urine  Vaginal | Control  RUTI  RTR_control  RTR_RUTI  RTR_RUTI_AB  Control  RUTI  RTR_control  RTR_RUTI  RTR_RUTI_AB  100  200  300  400  500  Urine  Vaginal |
| Abbreviations: AB=on antibiotic prophylaxis; RTR=renal transplant recipient; RUTI=recurrent urinary tract infection (defined as at least three UTIs in the past year). | |

**Figure S2B: Chao 1 alpha diversity and richness by type of sample and study group**

| **CHAO1** | | | | | | **RICHNESS** | | | | | |
| --- | --- | --- | --- | --- | --- | --- | --- | --- | --- | --- | --- |
| **Premenopausal** | **N per group** | **p-value** | **Postmenopausal** | **N per group** | **p-value*** | **Premenopausal** | **N per group** | **p-value** | **Postmenopausal** | **N per group** | **p-value*** |
| Urine: RUTI vs. controls | 18, 18 | 0.728 | Urine: RUTI vs. controls | 10, 20 | 0.239 | Urine: RUTI vs. controls | 18, 18 | 0.242 | Urine: RUTI vs. controls | 10, 20 | 0.157 |
|  |  |  | Urine: RTR_RUTI vs. RTR_controls | 6, 10 | 0.159 |  |  |  | Urine: RTR_RUTI vs. RTR_controls | 6, 10 | 0.233 |
|  |  |  | Urine: RTR_RUTI_AB vs. RTR_controls | 4, 10 | 0.090 |  |  |  | Urine: RTR_RUTI_AB vs. RTR_controls | 4, 10 | 0.120 |
| Vagina: RUTI vs. controls | 17, 15 | 0.358 | Vagina: RUTI vs. controls | 10, 20 | 0.370 | Vagina: RUTI vs. controls | 17, 15 | 0.716 | Vagina: RUTI vs. controls | 10, 20 | 0.164 |
|  |  |  | Vagina: RTR_RUTI vs. RTR_controls | 6, 10 | 0.515 |  |  |  | Vagina: RTR_RUTI vs. RTR_controls | 6, 10 | 0.515 |
|  |  |  | Vagina: RTR_RUTI_AB vs. RTR_controls | 4, 10 | 0.090 |  |  |  | Vagina: RTR_RUTI_AB vs. RTR_controls | 4, 10 | 0.077 |
| **Urine: pre- vs. postmenopausal controls** | | | | 18, 20 | <0.001 | **Urine: pre- vs. postmenopausal controls** | | | | 18, 20 | <0.001 |
| **Urine: pre- vs. postmenopausal RUTI cases** | | | | 18, 10 | <0.001 | **Urine: pre- vs. postmenopausal RUTI cases** | | | | 18, 10 | <0.001 |
| **Vagina: pre- vs. postmenopausal controls** | | | | 15, 20 | <0.001 | **Vagina: pre- vs. postmenopausal controls** | | | | 15, 20 | <0.001 |
| **Vagina: pre- vs. postmenopausal RUTI cases** | | | | 17, 20 | <0.001 | **Vagina: pre- vs. postmenopausal RUTI cases** | | | | 17, 20 | <0.001 |

* P-values are by Wilcoxon rank-sum tests.

**Figure S3: Unweighted and weighted UniFrac distances by type of sample and study group**

| **Premenopausal women: Unweighted UniFrac distances** | **Premenopausal women: Weighted UniFrac distances** |
| --- | --- |
| -0.75  -0.50  -0.25  0.00  0.25  0.50  -0.3  0.0  0.3  Axis.1 [19.9%]  Axis.2 [17%]  Body Site  Urine  Vaginal  Study Group  Control  RUTI | -0.5  0.0  0.5  -1.0  -0.5  0.0  0.5  Axis.1 [50.8%]  Axis.2 [24.7%]  Body Site  Urine  Vaginal  Study Group  Control  RUTI |
| **Postmenopausal women: Unweighted UniFrac distances** | **Postmenopausal women: Weighted UniFrac distances** |
| -0.50  -0.25  0.00  0.25  0.50  -0.6  -0.3  0.0  0.3  0.6  Axis 1 [9.6%]  Axis 2 [5.2%]  Body Site  Urine  Vaginal  Study Group  Control  RTR_control  RTR_RUTI  RTR_RUTI_AB  RUTI | -0.06  -0.03  0.00  0.03  0.06  -0.03  0.00  0.03  0.06  Axis 1 [23.3%]  Axis 2 [15.6%]  Body Site  Urine  Vaginal  Study Group  Control  RTR_control  RTR_RUTI  RTR_RUTI_AB  RUTI |
| Abbreviations: AB=on antibiotic prophylaxis; RTR=renal transplant recipient; RUTI=recurrent urinary tract infection (defined as at least three UTIs in the past year). | |

**Table S1: Mean relative abundances of bacterial groups and bacterial subgroups by study group**

| **Cells: mean RA in %** | **URINE SAMPLES** | | | | | | | **VAGINAL SAMPLES** | | | | | | |
| --- | --- | --- | --- | --- | --- | --- | --- | --- | --- | --- | --- | --- | --- | --- |
|  | **Premenopausal** | | **Postmenopausal** | | | | | **Premenopausal** | | **Postmenopausal** | | | | |
| **Bacterial groups** | **Control**  **(N=18)** | **RUTI**  **(N=18)** | **Control**  **(N=20)** | **RUTI**  **(N=10)** | **RTR control**  **(N=10)** | **RTR-RUTI**  **(N=6)** | **RTR-RUTI-AB (N=4)** | **Control**  **(N=15)** | **RUTI**  **(N=17)** | **Control**  **(N=20)** | **RUTI**  **(N=10)** | **RTR control**  **(N=10)** | **RTR-RUTI**  **(N=6)** | **RTR-RUTI-AB**  **(N=4)** |
| Lactobacilli | 44.66 | 37.44 | 10.50 | 2.96 | 18.03 | 12.20 | 17.19 | 66.31 | 59.83 | 10.29 | 4.46 | 23.75 | 17.03 | 16.75 |
| BV-anaerobes | 23.36 | 24.58 | 64.45 | 39.28 | 50.13 | 48.64 | 11.89 | 27.31 | 37.01 | 64.69 | 60.07 | 62.49 | 66.41 | 20.96 |
| Gram-positive uropathobionts | 4.63 | 8.05 | 12.80 | 21.77 | 9.30 | 12.15 | 17.82 | 4.89 | 1.02 | 14.93 | 17.01 | 4.99 | 5.21 | 13.12 |
| Gram-negative uropathobionts | 23.54 | 25.79 | 5.57 | 26.05 | 13.88 | 23.26 | 26.22 | 0.01 | 0.11 | 1.69 | 4.76 | 2.75 | 3.83 | 17.77 |
| Other bacteria | 3.82 | 4.14 | 6.69 | 9.94 | 8.66 | 3.76 | 26.89 | 1.48 | 2.02 | 8.40 | 13.70 | 6.02 | 7.51 | 31.40 |
| ***Lactobacillus* subgroups** |  |  |  |  |  |  |  |  |  |  |  |  |  |  |
| *Lactobacillus crispatus* | 20.91 | 16.00 | 1.18 | 0.42 | 0.11 | 0.00 | 2.51 | 28.07 | 24.09 | 1.52 | 0.22 | 0.23 | 0.02 | 1.16 |
| *Lactobacillus iners* | 9.37 | 14.75 | 5.40 | 1.67 | 9.55 | 6.66 | 0.10 | 21.49 | 23.65 | 4.75 | 2.84 | 11.17 | 10.28 | 0.04 |
| *Lactobacillus gasseri* | 0.00 | 0.63 | 0.00 | 0.00 | 0.00 | 0.00 | 0.00 | 0.00 | 0.59 | 0.00 | 0.00 | 0.00 | 0.00 | 0.00 |
| *Lactobacillus jensenii* | 6.77 | 1.16 | 2.95 | 0.01 | 0.34 | 0.07 | 0.07 | 5.81 | 3.36 | 2.99 | 0.01 | 0.32 | 0.38 | 0.02 |
| Other lactobacilli | 7.60 | 4.91 | 0.97 | 0.86 | 8.02 | 5.47 | 14.51 | 10.93 | 8.14 | 1.04 | 1.38 | 12.03 | 6.35 | 15.53 |
| **BV-anaerobes subgroups** |  |  |  |  |  |  |  |  |  |  |  |  |  |  |
| *Gardnerella vaginalis* | 10.28 | 13.15 | 19.68 | 14.68 | 16.22 | 19.65 | 0.46 | 14.81 | 22.56 | 15.39 | 13.29 | 17.46 | 21.02 | 0.07 |
| *Atopobium vaginae* | 2.97 | 2.14 | 4.02 | 3.15 | 5.13 | 0.00 | 0.05 | 6.27 | 6.11 | 4.45 | 4.22 | 7.17 | 0.06 | 0.01 |
| *Prevotella* | 3.20 | 2.87 | 10.12 | 9.29 | 7.88 | 9.34 | 1.99 | 1.13 | 0.59 | 9.82 | 18.37 | 11.65 | 16.68 | 6.76 |
| Other BV-anaerobes | 6.91 | 6.42 | 30.63 | 12.15 | 20.90 | 19.64 | 9.40 | 5.10 | 7.75 | 35.04 | 24.20 | 26.21 | 28.65 | 14.13 |
| **Gram-pos uropathobionts** |  |  |  |  |  |  |  |  |  |  |  |  |  |  |
| *Streptococcus agalactiae* | 1.47 | 4.99 | 3.13 | 0.07 | 4.14 | 0.00 | 0.00 | 4.12 | 0.04 | 5.69 | 0.08 | 1.90 | 0.01 | 0.00 |
| Other streptococci | 2.08 | 2.61 | 4.16 | 10.64 | 4.69 | 0.83 | 1.65 | 0.53 | 0.57 | 3.64 | 12.06 | 1.91 | 3.19 | 0.52 |
| *Actinotignum schaalii* | 0.00 | 0.00 | 0.36 | 1.17 | 0.22 | 10.28 | 0.39 | 0.00 | 0.00 | 0.26 | 2.83 | 0.33 | 1.45 | 1.56 |
| *Staphylococcus aureus* | 0.07 | 0.17 | 0.36 | 0.01 | 0.00 | 0.01 | 0.23 | 0.03 | 0.09 | 0.40 | 0.02 | 0.00 | 0.00 | 0.09 |
| *Aerococcus urinae* | 0.00 | 0.00 | 0.02 | 0.04 | 0.03 | 0.10 | 0.00 | 0.00 | 0.00 | 0.01 | 0.09 | 0.02 | 0.13 | 0.00 |
| *Enterococcus faecalis/faecium* | 0.00 | 0.00 | 0.00 | 0.00 | 0.00 | 0.00 | 0.01 | 0.00 | 0.00 | 0.00 | 0.00 | 0.00 | 0.00 | 0.01 |
| Other Gram-pos uropathobionts | 1.01 | 0.28 | 4.76 | 9.83 | 0.22 | 0.94 | 15.54 | 0.20 | 0.33 | 4.93 | 1.94 | 0.84 | 0.43 | 10.93 |
| **Gram-neg uropathobionts** |  |  |  |  |  |  |  |  |  |  |  |  |  |  |
| *Escherichia/Shigella* | 0.03 | 16.09 | 4.29 | 12.22 | 2.58 | 0.04 | 0.98 | 0.00 | 0.01 | 0.07 | 0.10 | 0.30 | 0.02 | 0.29 |
| *Klebsiella* | 0.00 | 0.00 | 0.05 | 8.33 | 7.87 | 22.00 | 14.63 | 0.00 | 0.00 | 0.00 | 0.16 | 0.07 | 0.16 | 0.54 |
| *Pseudomonas* | 16.56 | 9.19 | 0.00 | 0.02 | 0.00 | 0.00 | 8.30 | 0.00 | 0.03 | 0.01 | 0.06 | 0.00 | 0.00 | 16.49 |
| *Acinetobacter* | 4.71 | 0.05 | 0.47 | 4.64 | 0.00 | 0.00 | 0.28 | 0.00 | 0.00 | 0.08 | 2.73 | 0.00 | 0.00 | 0.01 |
| *Citrobacter* | 0.03 | 0.00 | 0.00 | 0.00 | 0.00 | 0.12 | 0.01 | 0.00 | 0.00 | 0.00 | 0.00 | 0.00 | 0.00 | 0.00 |
| *Enterobacter* | 0.05 | 0.00 | 0.00 | 0.01 | 0.09 | 0.01 | 0.20 | 0.00 | 0.00 | 0.00 | 0.00 | 0.00 | 0.00 | 0.00 |
| *Morganella* | 0.00 | 0.00 | 0.00 | 0.00 | 0.03 | 0.00 | 0.01 | 0.00 | 0.00 | 0.00 | 0.00 | 0.00 | 0.00 | 0.00 |
| *Serratia* | 0.00 | 0.00 | 0.00 | 0.00 | 0.00 | 0.00 | 0.05 | 0.00 | 0.00 | 0.00 | 0.00 | 0.00 | 0.00 | 0.01 |
| Other Gram-neg uropathobionts | 2.17 | 0.46 | 0.75 | 0.84 | 3.29 | 1.09 | 1.76 | 0.01 | 0.07 | 1.54 | 1.70 | 2.38 | 3.65 | 0.43 |
| **Other bacteria subgroups** |  |  |  |  |  |  |  |  |  |  |  |  |  |  |
| *Bifidobacterium* | 0.02 | 2.93 | 0.05 | 0.77 | 0.90 | 0.13 | 11.23 | 0.09 | 0.00 | 0.04 | 2.86 | 1.59 | 1.23 | 12.31 |
| *Corynebacterium* | 0.81 | 0.53 | 4.10 | 7.09 | 3.11 | 0.66 | 8.84 | 0.73 | 1.20 | 5.46 | 3.68 | 1.41 | 1.37 | 12.14 |
| Other bacteria | 2.99 | 0.67 | 2.54 | 2.08 | 4.66 | 2.96 | 6.82 | 0.67 | 0.82 | 2.90 | 7.16 | 3.02 | 4.92 | 6.95 |

Abbreviations: AB=antibiotic use (prophylactic); RA=relative abundance; RUTI=recurrent urinary tract infection (at least three times in last year); RTR=renal transplant recipient. P-values in next two tables.

**Table S2A: p-values for differences in mean relative abundances in urine samples between study groups**

| **URINE SAMPLES** | **Premenopausal** | | **Postmenopausal** | | | | | | **Postmenopausal vs Premenopausal** | | | |
| --- | --- | --- | --- | --- | --- | --- | --- | --- | --- | --- | --- | --- |
| **In cells: p-values** | **Control - RUTI** | | **Control - RUTI** | | **Control - RTR control** | | **RTR control - RTR RUTI** | | **PostM – PreM controls** | | **PostM– PreM RUTI** | |
| **Bacterial groups** | **p** | **adj p*** | **p** | **adj p*** | **p** | **adj p*** | **p** | **adj p*** | **p** | **adj p*** | **p** | **adj p*** |
| Lactobacilli | 0.406 | 0.643 | 0.871 | 1.000 | 0.286 | 0.776 | 0.436 | 0.734 | 0.000 | 0.000 | 0.011 | 0.022 |
| BV-anaerobes | 0.696 | 0.814 | 0.049 | 0.761 | 0.143 | 0.737 | 0.190 | 0.602 | 0.000 | 0.000 | 0.053 | 0.102 |
| Gram-positive uropathobionts | 0.079 | 0.361 | 0.444 | 0.974 | 0.812 | 0.968 | 0.796 | 1.000 | 0.051 | 0.090 | 0.005 | 0.017 |
| Gram-negative uropathobionts | 0.767 | 0.828 | 0.234 | 0.974 | 0.422 | 0.818 | 0.218 | 0.602 | 0.000 | 0.000 | 0.596 | 0.710 |
| Other bacteria | 0.013 | 0.117 | 0.594 | 0.974 | 0.812 | 0.968 | 1.000 | 1.000 | 0.276 | 0.415 | 0.007 | 0.019 |
| ***Lactobacillus* subgroups** |  |  |  |  |  |  |  |  |  |  |  |  |
| *Lactobacillus crispatus* | 0.452 | 0.643 | 0.726 | 0.992 | 0.126 | 0.737 | 0.957 | 1.000 | 0.006 | 0.017 | 0.116 | 0.212 |
| *Lactobacillus iners* | 0.987 | 0.987 | 0.736 | 0.992 | 0.875 | 0.968 | 0.135 | 0.602 | 0.039 | 0.073 | 0.006 | 0.018 |
| *Lactobacillus gasseri* | 0.345 | 0.612 | NA | NA | NA | NA | NA | NA | NA | NA | 0.530 | 0.670 |
| *Lactobacillus jensenii* | 0.349 | 0.612 | 1.000 | 1.000 | 0.260 | 0.776 | 0.728 | 1.000 | 0.484 | 0.558 | 0.977 | 0.979 |
| Other lactobacilli | 0.013 | 0.117 | 0.653 | 0.974 | 0.243 | 0.776 | 0.912 | 1.000 | 0.001 | 0.003 | 0.979 | 0.979 |
| **BV-anaerobe subgroups** |  |  |  |  |  |  |  |  |  |  |  |  |
| *Gardnerella vaginalis* | 0.825 | 0.856 | 0.660 | 0.974 | 0.983 | 0.983 | 0.796 | 1.000 | 0.539 | 0.578 | 0.898 | 0.979 |
| *Atopobium vaginae* | 0.362 | 0.612 | 0.513 | 0.974 | 0.573 | 0.912 | 0.143 | 0.602 | 0.403 | 0.504 | 0.540 | 0.670 |
| *Prevotella* | 0.438 | 0.643 | 0.835 | 1.000 | 0.475 | 0.865 | 1.000 | 1.000 | 0.017 | 0.040 | 0.004 | 0.017 |
| Other BV-anaerobes | 0.501 | 0.676 | 0.030 | 0.761 | 0.328 | 0.776 | 0.481 | 0.770 | 0.000 | 0.000 | 0.009 | 0.021 |
| **Gram-positive uropathobionts** |  |  |  |  |  |  |  |  |  |  |  |  |
| *Streptococcus agalactiae* | 0.616 | 0.793 | 0.545 | 0.974 | 0.928 | 0.983 | 0.301 | 0.602 | 0.038 | 0.073 | 0.133 | 0.229 |
| Other streptococci | 0.166 | 0.512 | 0.982 | 1.000 | 0.350 | 0.776 | 0.043 | 0.602 | 0.023 | 0.050 | 0.002 | 0.008 |
| *Actinotignum schaalii* | NA | NA | 0.144 | 0.974 | 0.692 | 0.933 | 0.280 | 0.602 | 0.000 | 0.000 | 0.000 | 0.000 |
| *Staphylococcus aureus* | 0.237 | 0.583 | 0.438 | 0.974 | 0.105 | 0.737 | 0.267 | 0.602 | 0.421 | 0.505 | 0.789 | 0.906 |
| *Aerococcus urinae* | NA | NA | 0.981 | 1.000 | 0.076 | 0.737 | 0.168 | 0.602 | 0.000 | 0.000 | 0.001 | 0.006 |
| *Enterococcus faecalis/faecium* | NA | NA | 0.551 | 0.974 | 0.525 | 0.903 | 0.078 | 0.602 | 0.370 | 0.504 | NA | NA |
| Other Gram-pos uropathobionts | 0.299 | 0.612 | 0.274 | 0.974 | 0.248 | 0.776 | 0.043 | 0.602 | 0.082 | 0.129 | 0.008 | 0.019 |
| **Gram-negative uropathobionts** |  |  |  |  |  |  |  |  |  |  |  |  |
| *Escherichia/Shigella* | 0.314 | 0.612 | 0.499 | 0.974 | 0.869 | 0.968 | 0.297 | 0.602 | 0.712 | 0.737 | 0.934 | 0.979 |
| *Klebsiella* | NA | NA | 0.402 | 0.974 | 0.063 | 0.737 | 0.241 | 0.602 | 0.002 | 0.005 | 0.001 | 0.006 |
| *Pseudomonas* | 0.171 | 0.512 | 1.000 | 1.000 | 0.378 | 0.781 | 0.124 | 0.602 | 0.000 | 0.000 | 0.000 | 0.001 |
| *Acinetobacter* | 0.197 | 0.532 | 0.527 | 0.974 | 0.744 | 0.961 | 0.284 | 0.602 | 0.054 | 0.090 | 0.333 | 0.449 |
| *Citrobacter* | 0.163 | 0.512 | 0.629 | 0.974 | 0.254 | 0.776 | 0.518 | 0.790 | 0.512 | 0.568 | 0.182 | 0.268 |
| *Enterobacter* | 0.080 | 0.361 | 0.527 | 0.974 | 0.315 | 0.776 | 0.898 | 1.000 | 0.774 | 0.774 | 0.182 | 0.268 |
| *Morganella* | NA | NA | 0.157 | 0.974 | 0.047 | 0.737 | 0.584 | 0.850 | NA | NA | 0.182 | 0.268 |
| *Serratia* | NA | NA | NA | NA | NA | NA | 0.368 | 0.693 | NA | NA | NA | NA |
| Other Gram-neg uropathobionts | 0.064 | 0.361 | 1.000 | 1.000 | 0.619 | 0.913 | 0.912 | 1.000 | 0.346 | 0.495 | 0.322 | 0.449 |
| **Other bacteria subgroups** |  |  |  |  |  |  |  |  |  |  |  |  |
| *Bifidobacterium* | 0.724 | 0.814 | 0.307 | 0.974 | 0.672 | 0.933 | 0.102 | 0.602 | 0.013 | 0.033 | 0.010 | 0.022 |
| *Corynebacterium* | 0.666 | 0.814 | 0.945 | 1.000 | 0.983 | 0.983 | 0.393 | 0.699 | 0.002 | 0.007 | 0.002 | 0.009 |
| Other bacteria | 0.001 | 0.020 | 0.417 | 0.974 | 0.588 | 0.912 | 0.912 | 1.000 | 0.393 | 0.504 | 0.001 | 0.008 |

*Adjusted Wilcoxon rank-sum p-values (Benjamin-Hochberg correction) were listed when at least one unadjusted p-value in a column was below 0.05.

**Figure S2B: p-values for differences in mean relative abundances in vaginal samples between study groups**

| **VAGINAL SAMPLES p-values** | **Premenopausal** | | **Postmenopausal** | | | | | | **Postmenopausal vs premenopausal** | | | |
| --- | --- | --- | --- | --- | --- | --- | --- | --- | --- | --- | --- | --- |
|  | **Control - RUTI** | | **Control - RUTI** | | **Control - RTR control** | | **RTR control - RTR RUTI** | | **PostM–PreM controls** | | **PostM– PreM RUTI** | |
| **Bacterial groups** | **p** | **adj p*** | **p** | **adj p*** | **p** | **adj p*** | **p** | **adj p*** | **p** | **adj p*** | **p** | **adj p*** |
| Lactobacilli | 0.892 | 0.959 | 0.835 | 0.973 | 0.267 | 0.825 | 0.684 | 0.818 | 0.000 | 0.000 | 0.001 | 0.004 |
| BV-anaerobes | 0.892 | 0.959 | 0.501 | 0.973 | 0.475 | 0.917 | 0.353 | 0.662 | 0.012 | 0.019 | 0.251 | 0.325 |
| Gram-positive uropathobionts | 0.363 | 0.959 | 0.417 | 0.973 | 0.475 | 0.917 | 0.971 | 0.971 | 0.003 | 0.007 | 0.000 | 0.002 |
| Gram-negative uropathobionts | 0.110 | 0.955 | 0.908 | 0.973 | 0.948 | 0.983 | 0.393 | 0.662 | 0.000 | 0.000 | 0.001 | 0.004 |
| Other bacteria | 0.922 | 0.959 | 0.627 | 0.973 | 0.983 | 0.983 | 0.143 | 0.662 | 0.000 | 0.000 | 0.001 | 0.004 |
| **Bacterial subgroups** |  |  |  |  |  |  |  |  |  |  |  |  |
| *Lactobacillus crispatus* | 0.435 | 0.959 | 0.559 | 0.973 | 0.978 | 0.983 | 0.690 | 0.818 | 0.001 | 0.003 | 0.164 | 0.243 |
| *Lactobacillus iners* | 0.323 | 0.959 | 0.345 | 0.973 | 0.582 | 0.981 | 0.569 | 0.764 | 0.053 | 0.073 | 0.004 | 0.010 |
| *Lactobacillus gasseri* | 0.590 | 0.959 | NA | NA | NA | NA | NA | NA | 0.106 | 0.134 | 0.505 | 0.580 |
| *Lactobacillus jensenii* | 0.610 | 0.959 | 0.859 | 0.973 | 0.679 | 0.981 | 0.545 | 0.764 | 0.430 | 0.520 | 0.647 | 0.692 |
| Other lactobacilli | 0.093 | 0.955 | 0.179 | 0.767 | 0.322 | 0.825 | 0.597 | 0.764 | 0.000 | 0.001 | 0.419 | 0.500 |
| **BV-anaerobes subgroups** |  |  |  |  |  |  |  |  |  |  |  |  |
| *Gardnerella vaginalis* | 0.470 | 0.959 | 0.524 | 0.973 | 0.878 | 0.981 | 0.315 | 0.662 | 0.629 | 0.729 | 0.149 | 0.231 |
| *Atopobium vaginae* | 0.592 | 0.959 | 0.981 | 1.000 | 0.839 | 0.981 | 0.517 | 0.764 | 0.825 | 0.886 | 0.411 | 0.500 |
| *Prevotella* | 0.984 | 0.984 | 0.153 | 0.767 | 0.880 | 0.981 | 0.739 | 0.845 | 0.000 | 0.000 | 0.000 | 0.002 |
| Other BV-anaerobes | 0.861 | 0.959 | 0.501 | 0.973 | 0.267 | 0.825 | 0.971 | 0.971 | 0.000 | 0.000 | 0.004 | 0.010 |
| **Gram-positive uropathobionts** |  |  |  |  |  |  |  |  |  |  |  |  |
| *Streptococcus agalactiae* | 0.627 | 0.959 | 0.748 | 0.973 | 0.836 | 0.981 | 0.024 | 0.662 | 0.104 | 0.134 | 0.070 | 0.114 |
| Other streptococci | 0.608 | 0.959 | 0.627 | 0.973 | 0.846 | 0.981 | 0.218 | 0.662 | 0.002 | 0.004 | 0.001 | 0.004 |
| *Actinotignum schaalii* | NA | NA | 0.032 | 0.659 | 0.118 | 0.825 | 0.970 | 0.971 | 0.000 | 0.000 | 0.000 | 0.000 |
| *Staphylococcus aureus* | 0.817 | 0.959 | 1.000 | 1.000 | 0.266 | 0.825 | 0.169 | 0.662 | 0.749 | 0.835 | 0.548 | 0.607 |
| *Aerococcus urinae* | NA | NA | 0.846 | 0.973 | 0.166 | 0.825 | 0.432 | 0.691 | 0.013 | 0.019 | 0.018 | 0.036 |
| *Enterococcus faecalis/faecium* | NA | NA | NA | NA | 0.179 | 0.825 | 0.584 | 0.764 | NA | NA | NA | NA |
| *Other Gram-pos uropathobionts* | 0.921 | 0.959 | 0.317 | 0.973 | 0.198 | 0.825 | 0.393 | 0.662 | 0.011 | 0.019 | 0.016 | 0.033 |
| **Gram-negative uropathobionts** |  |  |  |  |  |  |  |  |  |  |  |  |
| *Escherichia/Shigella* | 0.214 | 0.959 | 0.379 | 0.973 | 0.760 | 0.981 | 0.104 | 0.662 | 0.011 | 0.019 | 0.061 | 0.107 |
| *Klebsiella* | NA | NA | 0.044 | 0.659 | 0.430 | 0.917 | 0.313 | 0.662 | 0.013 | 0.019 | 0.000 | 0.002 |
| *Pseudomonas* | 0.195 | 0.959 | 0.366 | 0.973 | 0.334 | 0.825 | 0.168 | 0.662 | 0.880 | 0.901 | 0.830 | 0.858 |
| *Acinetobacter* | 0.071 | 0.955 | 0.517 | 0.973 | 0.016 | 0.457 | 0.168 | 0.662 | 0.010 | 0.019 | 0.943 | 0.943 |
| *Citrobacter* | NA | NA | 0.157 | 0.767 | NA | NA | 0.368 | 0.662 | NA | NA | 0.211 | 0.285 |
| *Enterobacter* | 0.333 | 0.959 | 0.154 | 0.767 | 0.525 | 0.951 | 0.368 | 0.662 | 0.901 | 0.901 | 0.062 | 0.107 |
| *Morganella* | NA | NA | NA | NA | NA | NA | 0.368 | 0.662 | NA | NA | NA | NA |
| *Serratia* | NA | NA | 0.157 | 0.767 | NA | NA | 0.368 | 0.662 | NA | NA | 0.211 | 0.285 |
| Other Gram-neg uropathobionts | 0.860 | 0.959 | 0.908 | 0.973 | 0.860 | 0.981 | 0.796 | 0.878 | 0.000 | 0.000 | 0.000 | 0.002 |
| **Other bacteria subgroups** |  |  |  |  |  |  |  |  |  |  |  |  |
| *Bifidobacterium* | 0.805 | 0.959 | 0.868 | 0.973 | 0.341 | 0.825 | 0.241 | 0.662 | 0.000 | 0.001 | 0.007 | 0.016 |
| *Corynebacterium* | 0.663 | 0.959 | 0.764 | 0.973 | 0.619 | 0.981 | 0.218 | 0.662 | 0.004 | 0.008 | 0.010 | 0.022 |
| Other bacteria | 0.830 | 0.959 | 0.729 | 0.973 | 0.231 | 0.825 | 0.280 | 0.662 | 0.000 | 0.000 | 0.002 | 0.005 |

*Adjusted Wilcoxon rank-sum p-values (Benjamin-Hochberg correction) were listed when at least one unadjusted p-value in a column was below 0.05.

**Figure S4: Relative abundances of bacterial groups in individual samples**

| 1. **Premenopausal women – urine samples** | | | | | |
| --- | --- | --- | --- | --- | --- |
| **Controls (N=18)** | | **RUTI (N=18)** | | | |
|  | | | | | |
| 1. **Premenopasual women – vaginal samples** | | | | | |
| **Controls (N=15)** | | **RUTI (N=17)** | | | |
|  | | | | | |
| 1. **Postmenopausal women – urine samples** | | | | | |
| **Controls (N=20)** | **RUTI (N=10)** | | **RTR controls (N=10)** | **RTR RUTI (N=6)** | **RTR RUTI AB (N=4)** |
|  | | | | | |
| 1. **Postmenopausal women – vaginal samples** | | | | | |
| **Controls (N=20)** | **RUTI (N=10)** | | **RTR controls (N=10)** | **RTR RUTI (N=6)** | **RTR RUTI AB (N=4)** |
|  | | | | | |
| Lactobacilli; BV-anaerobes; Gram-positive uropathobionts; Gram-negative uropathobionts; Other bacteria | | | | | |

Abbreviations: AB=on antibiotic prophylaxis; BV=bacterial vaginosis; RTR=renal transplant recipient; RUTI=recurrent urinary tract infection (defined as at least three UTIs in the past year). Each column represents one unique sample.

**Figure S5: Relative abundances in premenopausal women by ethnicity and RUTI status**

| 1. **Premenopausal women – urine samples** | | | |
| --- | --- | --- | --- |
| **Controls SSA (N=5)** | **Controls Dutch (N=13)** | **RUTI SSA (N=5)** | **RUTI Dutch (N=13)** |
|  | | | |
| 1. **Premenopasual women – vaginal samples** | | | |
| **Controls SSA (N=5)** | **Controls Dutch (N=10)** | **RUTI SSA (N=5)** | **RUTI Dutch (N=12)** |
|  | | | |
| Lactobacilli; BV-anaerobes; Gram-positive uropathobionts; Gram-negative uropathobionts; Other bacteria | | | |

| **Urine** | **SSA controls** | **Dutch controls** | **SSA RUTI** | **Dutch RUTI** | **SSA total** | **Dutch total** |
| --- | --- | --- | --- | --- | --- | --- |
| **Lactobacilli** | 40.89 | 44.32 | 15.88 | 48.80 | 28.38 | 46.56 |
| **BV-anaerobes** | 39.44 | 18.13 | 35.30 | 16.70 | 37.37 | 17.41 |
| **Gram-positive uropathobionts** | 7.93 | 1.64 | 1.26 | 11.55 | 4.60 | 6.60 |
| **Gram-negative uropathobionts** | 7.73 | 31.52 | 34.76 | 22.18 | 21.24 | 26.85 |
| **Other bacteria** | 40.27 | 4.39 | 12.80 | 0.78 | 8.41 | 2.58 |
| **Vagina** |  |  |  |  |  |  |
| **Lactobacilli** | 44.21 | 77.36 | 29.41 | 73.67 | 36.81 | 75.51 |
| **BV-anaerobes** | 39.80 | 21.07 | 64.70 | 24.42 | 52.25 | 22.74 |
| **Gram-positive uropathobionts** | 12.52 | 1.07 | 0.46 | 1.28 | 6.49 | 1.17 |
| **Gram-negative uropathobionts** | 0 | 0 | 0.13 | 0.11 | 0.07 | 0.06 |
| **Other bacteria** | 3.46 | 0.49 | 5.31 | 0.53 | 4.39 | 0.51 |

In the bar chart, each column represents one unique sample. In the table, cells contain mean RA in %.

Abbreviations: BV=bacterial vaginosis; RUTI=recurrent urinary tract infection (defined as at least three UTIs in the past year); RA=relative abundance; SSA=sub-Saharan African descent.

**Figure S6: Relative abundances of *Lactobacillus* species in individual samples**

| **Premenopausal women – urine samples** | | **Premenopausal women – vaginal samples** | |
| --- | --- | --- | --- |
| **Controls (N=18)** | **RUTI (N=18)** | **Controls (N=15)** | **RUTI (N=17)** |
|  |  |  |  |
| **Postmenopausal women (non-RTR) – urine samples** | | **Postmenopausal women (non-RTR) – vaginal samples** | |
| **Controls (N=20)** | **RUTI (N=10)** | **Controls (N=20)** | **RUTI (N=10)** |
|  |  |  |  |
| *L. crispatus*; *L. iners*; *L. gasseri*; *L. jensenii*; Other or unresolved lactobacilli | | | |

| **Postmenopausal RTR – urine samples**  **(RTR controls 10, RTR RUTI 6, RTR RUTI using antibiotic prophylaxis 4)** | **Postmenopausal RTR – vaginal samples**  **(RTR controls 10, RTR RUTI 6, RTR RUTI using antibiotic prophylaxis 4)** |
| --- | --- |
|  |  |
| *L. crispatus*; *L. iners*; *L. gasseri*; *L. jensenii*; Other or unresolved lactobacilli | |

Abbreviations: AB=on antibiotic prophylaxis; RTR=renal transplant recipient; RUTI=recurrent urinary tract infection (defined as at least three UTIs in the past year). Each bar represents one unique sample. Unresolved lactobacilli could only be identified at genus level, not at species level.
